# A History of Epidemic Cholera in Berlin in the Year 1853
*We offer no apology for putting this important official document before our readers in an English dress. The name of Dr. Müller is alone a warranty for the act. Mr. West's translation is a faithful representation of the original. It will be interesting to compare this Report with the Reports issued by the Board of Health here, on the Progress and Treatment of the Cholera in London during the late outbreaks.—*Editor*.


**Published:** 1855-09

**Authors:** R. U. West


### PROGRESS OF EPIDEMICS.

### A HISTORY OF EPIDEMIC CHOLERA IN BERLIN IN THE YEAR 1853.*

By Dr. Müller. Translated from the German by R. U. West, Esq.

The cholera-epidemic of the year 1852, came to an end in Berlin with the close of the year. This was not the case in the eastern provinces of Prussia, where, notwithstanding the cold of winter, the disease continued to occur here and there. Whether, therefore, between the spring and summer of 1853 the cholera was entirely extinguished in Prussia, is, to us at least, quite unknown. We only know that information respecting its existence was not made public, until, at the time of the summer heats cholera cases were again announced from Dantzic, Stettin, and other places.

In Berlin, the first cholera case of the year 1853, occurred on the 7th of August. It attacked a young woman, who fell sick in Rosenthaler-street, probably in consequence of infection (her master had shortly before returned home from Swinemonde, where the cholera was raging), and died in the Charity Hospital. A few days later occurred the equally fatal cases of a sailor, who lay in a boat at the ship-building pier, and of a widow belonging to the wealthier classes, in a house, No. 18, Albert-street. Further cases occurred singly, until the last third of August, when they began to take place every day, and there was no longer any doubt respecting the epidemic character of the disease.

Science has hitherto failed to enlighten us as to whether any, and what, connexion is to be found between cholera-epidemics and the meteorological phenomena preceding and accompanying them. Nevertheless, it seems to be necessary, in giving the history of an epidemic, not to leave unnoticed the meteorological observations of the period.

According to Dr. Schultz's observations, the quantity of ozone in the atmosphere was the greatest in precisely those months in which the cholera was least prevalent in the doctor's professional district. The average of ozone in August, with 18 cases, was 2.677; in September, with 121 cases, 2.900; in October, with 31 cases, 1.258; and in November, with 5 cases, only 0.366. This physician remarks further, that, when we look upon the cholera, or, in fact, any diseases whose origin can be considered to have

---

* We offer no apology for putting this important official document before our readers in an English dress. The name of Dr. Müller is alone a warranty for the act. Mr. West's translation is a faithful representation of the original. It will be interesting to compare this Report with the Reports issued by the Board of Health here, on the Progress and Treatment of the Cholera in London during the late outbreaks.—*Editor.*

any relation to meteorological circumstances, as *consequences* of these circumstances, and do not look for a *simultaneous* appearance of the disease, but rather one in which effect follows cause, then we shall perceive that the cholera-epidemic of this year establishes in a remarkable manner Professor August's views respecting its connexion with atmospheric humidity. In August, September, and October, according to his observations, the greatest comparative humidity prevailed, and this decreased from its maximum of August, as did also the cholera in every consecutive month. Upon the comparatively wettest month, August, followed the sickliest month September; and on the fall in the atmospheric humidity which took place in September, and still more in October, followed a corresponding falling off in the number of the sick in October and November.

*Meteorological Observations for* 1853.

| Month. | Average of barometer in Parisian lines. | Average of thermom., Fahr. | Average humidity in per centages. | Average of the weather. | Prevailing winds. | Remarks. |
|---|---|---|---|---|---|---|
| Jan. | 334·41 | 37·5 | 85 | Dull, snow | s. and s.w. | 1 thunder- |
| Feb. | 331·58 | 35·5 | 83 | Dull, cldy., snow | E. and N.E. | [storm. |
| Mar. | 335·96 | 35·5 | 79 | Dull and cloudy | S.W. N. N.W. | |
| April | 334·14 | 41·9 | 75 | Dull and cloudy | W. N.W. S.E. | |
| May | 335·57 | 59·4 | 62 | Bright, cloudy | W. N.E. E. | |
| June | 334·27 | 64·9 | 70 | Brigt., cldy, rain | W. N.W. N. | 11 storms. |
| July | 335·64 | 66·7 | 75 | Bright, cloudy | W. S. S.W. | 8 storms. |
| Aug. | 335·61 | 62·8 | 75 | Bright, cloudy | S.W. W. | 2 storms. |
| Sept. | 335·60 | 57·2 | 73 | Brigt., cldy., rain | S.W. W. | |
| Oct. | 334·94 | 49·2 | 81 | Bright, cloudy | W. S.E. | |
| Nov. | 338·38 | 37·0 | 85 | Dull, fogs | E. S.E. | |
| Dec. | 236·5 | 37·7 | 87 | Brigt., dull, fogs | E. N.E. | |
| Aver. of Year | 335.22 | 46·2 | 77 | Bright, cloudy | E. W. N.W. N.E. | Highest temperature, 26·1 Réaum. Lowest 14·0 |

Next after the attendant meteorological circumstances, it is necessary, in the study of epidemics, to point out the other morbid conditions from which the epidemic may have developed itself, as well as those to which it may give rise.

In January, 1853, the prevailing morbid constitution was decidedly catarrhal; in February, catarrho-rheumatic. Measles were common; inflammatory diseases and small-pox less so, while gastric and intermittent fevers were very rare. A tendency to hæmorrhages was surprisingly frequent. Diarrhœa was not met with. This morbid constitution continued throughout March, hæmorrhages being likewise frequent, and a great liability to abortions with uterine hæmorrhage. Intermittent fevers showed themselves here and there; gastric fevers continued rare: on the other hand diarrhœas were observed to be frequent. The following month presented an increase in the rheumatic character of diseases, and, along with it, a not unfrequent tendency to gastric derangement. Measles and small-pox continued, as likewise diarrhœa. Intermittent fevers were rare; the tendency to hæmorrhages had ceased. Boils were remarkably frequent. In May the prevailing diseases were catarrho-gastric; intermittent fevers were frequent, still more so malignant measles. Diarrhœa with vomiting was present not unfrequently. The same morbid constitution, with prevalence of the gastric element, characterised the month of June. Diarrhœas, with and without vomitings, were observed, but not frequently, and they were very manageable. Intermittent fevers had become frequent. Hooping-cough, frequently complicated with bronchitis, appeared as an epidemic. Small-pox and urticaria were observed with tolerable frequency. In July the gastric character of the morbid constitution was still more distinctly marked. Diarrhœa became more frequent, but of a mild kind, and attacking chiefly children in the first years of their life. Hooping-cough and intermittent fevers continued to prevail. The gastric character of the prevailing diseases maintained itself in the following month, August. Diarrhœas, with and without vomitings, became more frequent, but it was only in the beginning of the month that solitary cases of the former made themselves known as true cholera cases, while in the last third of the month the choleraic character of these diseases became more decided. In September, in which month likewise the gastric continued to be the prevailing element, the cholera had decidedly established itself as an epidemic, but we still had also typhus and hooping-cough. Inflammatory diseases, hæmorrhages and scarlatina, occurred sporadically. Along with the subsidence of the cholera-epidemic in October and November, the catarrhal character of diseases showed itself again; and until the end of the year, sometimes with inflammatory complications, the organs of respiration suffered from diseases of this kind.

On the whole, therefore, it was manifest that the catarrhal morbid constitution which prevailed in the beginning of the year gradually passed over into the gastric, this decidedly predominating from August to October (the period of the cholera epidemic), and then suddenly again vacating the field in favour of the catarrhal class of diseases.

Of not less importance than the general etiological influences of the epidemic—nay, of far greater—is it to inquire into the causes which may have been followed by sporadic cases. Ten official physicians of Berlin were required to direct their especial attention to the causes of sporadic cases; and the following is an account of what they observed. Dr. Schrœder, physician to the second medical district, assigned, as obvious causes of the disease, in nine cases, cold; in five cases, want of food; in two cases, cold and hunger; in fourteen cases, diarrhœa; in thirteen cases, depressing mental affections, such as fright, fear, sorrow, and vexation; in one case, an irregular life, and a total neglect of medical aid; in one case, drunkenness. Two cases occurred in unhealthy cellar-residences. One case occurred in an individual who came from out of doors into a house in which there were already several sick people. In seven cases, the sufferers had in all probability exposed themselves to infection by visits to cholera patients. In ten cases there was no assignable cause. With reference to the cases which occurred in the district situated in the second medical circuit, Dr. Schrœder remarks:—

The cause of the small number of cases in this quarter of the town is to be found in the greater opulence, and in the nature of the occupation of its inhabitants. The majority belong to the mercantile classes, and are either never exposed to the influences of the weather, or are in a position to protect themselves from them; they quickly provide themselves with medical aid, and never experience want in the necessaries of life. The number of mechanics living in this district is insignificant, and there are but few factories and industrial institutions to be found in it. The wide straight streets, crossing one another at right angles, provide a good ventilation. The injurious exhalations of the gutters are done away with by means of newly-constructed cesspools. The cleanliness of the streets is further provided for by the construction of a number of public urinaries. Several large open squares, in part planted with trees, exercise a beneficial influence in the purification of the air. Most of the houses are new, and built on an uniform plan. Their courts are roomy, cleanly, dry, and light, and not too thickly inhabited. The water for drinking is excellent. All the cases of sickness, with two exceptions, occurred in streets running north and south; the direction of the wind in September was chiefly south-west and west.

The same physician says of the seventh municipal division, which also is situated in the second medical circuit:—

The seventh division, which is the oldest part of Berlin and is situated between the two arms of the Spree, contains a great number of more or less damp dwelling places, especially on the heights. The water in most of the wells on the *Friedrichsgracht*, in the Fish-bridge, often smells very bad in the summer, looks muddy, is full of organised substances, and is useless for cooking, to say nothing of drinking. Here dwell a great number of persons belonging to the working classes, living chiefly in small rooms, and most of them in impoverished circumstances. Where poverty in damp, dark, small low dwellings is provided with bad water, there will be circumstances enough cooperating in producing and giving potency to diseases, to which in Fischerstrasse must be added the deleterious influence of certain trades, the carrying on of which impregnates the atmosphere with animal particles. In Fischerstrasse (Fish-street) No. 39 is a manufactory of glue, a tannery, and a curriery; and at No. 40, a morocco-leather factory. It is, therefore, not to be wondered at, if every epidemic finds the greatest number of victims in these and the neighbouring houses. In the houses Nos. 38, 39, 40, and 42, fourteen persons were attacked with the epidemic, and only three of them recovered. The atmosphere in the courts of these houses is impregnated with ill smelling substances, the prejudicial influence of which appears the more plainly when one considers the surrounding circumstances which interfere with a free circulation of air. Fish-street itself is not straight, but has eight different directions. The arm of the Spree which supplies the water for carrying on the above-named trades, flows up against the backs of the houses Nos. 37 to 42, the river flowing under the first five houses of the Fish-bridge, in which there were no cases of cholera during this epidemic. The winds coming from this side, therefore, find themselves stopped by these five houses, while towards the north are situated the houses Nos. 14 to 18 of the mill-dam, which towards the north side of the hinder houses of Fish-street form an acute angle with the houses of Fish-bridge. The winds, therefore, which come from this side find an opposition in these five houses, while towards the north side are found the houses 14 to 18, and towards the same side, the back houses of Fish-street form an acute angle with those of the Fish-bridge. The police order of the 5th of April, 1796, according to which those trades, the carrying on of which is accompanied by noxious effluvia, shall be carried on only in situations where a free current of air is not impeded by close buildings, is made nugatory by a rescript of the 21st August, 1798, which orders that in cases of transfer no limitation of trade privileges shall take place where pecuniary loss is likely to result. For this last would undoubtedly be the case in almost every instance, so long as no concessions are granted for the establishment of new businesses, and the value of the old establishments is thereby enhanced. As it is notorious that in these houses cholera cases have been very virulent, while experience has shown that an atmosphere impregnated with animal substances, especially where there are no free currents of air, aggravates the intensity of the disease, it must on every account be in the universal interest of the state policy to make an alteration in the law. I must here mention two other nuisances. Many of the houses in the seventh quarter have no privy, so that the expression "to run to the bridge" is equivalent to saying *one is attacked with diarrhœa,* for there is a public privy under the island bridge to which those who have not privies of their own can go. Perhaps there may not be everywhere room for privies ; but this a question to be decided by an architect. A second inconvenience is found in the fact that two persons frequently occupy but one bed.

Dr. Arnd, physician to the third medical district, asserts : The inquiry into the causes of the disease, even in the limited circle of a medical district, meets with invincible difficulties, if one is inclined to give credit to the assertions or opinions of the patients or their friends. Numberless contradictions, he continues, show that those giving information respecting the causes of the disease are just in as much uncertainty as those who seek it; or that they, jumping at once at a conclusion, here, as everywhere else, assign a catching cold as the cause of the disease. Communications respecting errors in diet as having induced the disease, appear to be undeserving of attention, when it is pretended that it has arisen from the use of aliments which had been in ordinary use by the patients without any bad consequences during the epidemic. My inquiries have failed in showing that the causes of the disease are independent of local influences. It can only be said that when all the circumstances are put together, local influences can no more be accused, but rather it would seem that the disease may arise under local influences seemingly the most favourable for health. Even when the example was produced that in a certain house, between the 25th of August and the 3rd of September, there occurred five cases, followed by one on the 15th of September. At the first moment the locality seemed of importance, but on inquiry it was found that local circumstances had nothing to do with this concurrence of five cases, but rather that there had been a number of accidental circumstances concurring to throw these patients together—circumstances to which in a large town like Berlin, where there are so many varieties and conditions of life, even the inhabitants of a single house are frequently exposed. A more sure proof that cases of sickness have not been heaped together by accident is afforded in my opinion by the constancy and regularity of their occurrence. If we apply this rule to the Wall-street, we there find occasional causes in operation in a greater proportion than in any other street of the third medical district. On the 30th August the first case was announced there, and on the 28th of September the last; and in the interval there were thirty-six cases in nineteen different houses. But it is remarkable, that while the cholera in Berlin generally first broke out on the 7th of August, it was not until the disease had lasted twenty-three days that the first case occurred in this street, and consequently after a rather considerable duration of the epidemic, and that it suddenly disappeared there on the 28th of September, while there were still cases of sickness occurring in other streets of the district in October. Perhaps this may be explained by the circumstance that the conditions for the origination of the disease are for the most part imported; and, therefore, in course of time find the most favourable places, but these also after some time exhaust themselves, as shewn by the sudden appearance and disappearance of the disease in that street. When, as already observed, a certain sum of accidental circumstances does not appear to me to furnish a sufficient cause why a disproportionate number of cases should have occurred in Wall-street, then I feel bound to look upon local influences as having induced them. Thus, if I compare Wall-street with Dresden-street, which is quite as populous, and the population of which, on the whole, does not partake of more favourable conditions of life than those of Wall-street, then the difference in the number of the sick appears to be extraordinarily great, and I can only attribute this difference, so much to the disadvantage of Wall-street, to an accumulation of more unfavourable local circumstances. Wall-street is constantly damp, dirty, the houses narrow, with small yards, the atmosphere in them being at all times of the year of a musty disagreeable smell. On the other hand, the streets to which the fresh air comes from the Köpnick-fields are altogether remarkable for the small number of cholera cases occurring in them. With regard to the bad drinking-water which the wells in New Cologne and partially in Wall-street furnish, and whose prejudicial influence at the time of the cholera-pest has been pointed out, I would remark that I look upon it as of no importance for Berlin generally, as well as for those streets in particular, as the opportunity of procuring better water is always at hand, and is also made use of.

Dr. Koblanck, physician to the fourth district, in the whole of which only twenty-three persons were attacked (in one family, four; in another, three; in a third, two; and for the rest, only one in one house and one family), remarks, with respect to etiology, that in none of the single cases was there any decided proof of infection, and that almost always errors in diet and neglected diarrhœas appeared to have been the causes.

Health Councillor Dr. Nagel, physician to the sixth district, in which seventy-four cases occurred (four, in five houses; three, in one house; two, in nine houses; and the remaining cases single), remarks that while the occurrence of several cases in a single house is often accidental, it is at other times unquestionably attributable to over-crowding, deficient ventilation, and other local conditions not yet satisfactorily made out, such as damp floors, bad water for drinking, and the like, and lastly, at other times the occurrence of a number of cases rapidly one after another in the same houses must be attributed to contagion, either immediate, through contact, or mediate, through some physical influence. To this class would appear to belong in part the cases which occurred in Potsdam-street, No. 130,* as well as that of a female who, dwelling in Sebastian-street, went to work in Lower-wall-street, No. 23, where two days before there had been a case of cholera, and there fell sick and died. In the same way Dr. Nagel explains most of the other cases where two or more individuals in one and the same houses were attacked in quick succession.

Dr. Perle, physician to the seventh district, in which one hundred and seventy-two individuals fell ill, says, respecting the probable causes: In this year, also, the first cases appear again to have occurred in the boats coming into the town, and most probably were brought from Stettin to Berlin. If one reflects that those dwellings which were nearest the water, such as the Ship-builder's-pier on the Spree, and Albert-street near the Panke, polluted with mud and

* On these cases it must not be overlooked, that in earlier cholera-epidemics cases occurred here, and that this house towards the north lies hidden behind high trees, and has no chambers opening to the other quarters, the solitary chamber opening to the north being always surprisingly cold even in the warmest weather.

dirt, as well as a part of Charles-street, the foundations of whose houses rest on moor and bog, were attacked with the greatest severity, I cannot help attributing an essential pernicious influence to the water with its gaseous exhalations, in the production of cholera miasms. In addition, errors in diet, and chills caught, must have played secondary parts as exciting causes.

Dr. Schultz, physician to the eighth medical district, in which there were one hundred and seventy-five cases, says—There was no decided continuous progress of the epidemic. It occurred indifferently in houses lying at a distance from one another, stepping over the adjoining ones. In many families, several individuals were attacked; in some instances, all together; in others, one after another; while in other families the disease was satisfied with a single victim. No description of dwellings enjoyed complete exemption. In cellars and attics, as well as in intermediate floors; in such as were kept clean and wholesome, as well as in the contrary, the disease would break out. Persons who were immediately in contact with the sick were exempt, in fact, the majority of such, while others, in whose cases no direct communication with the sick could be proved, were attacked by the pestilence and carried off. In an overwhelming majority of the cases, gastric symptoms of greater or less severity were observed to have been present before the outbreak of the cholera; but these also were wanting in a few solitary instances. The use of potatoes appeared as a prominent exciting cause in many instances, but by no means as the only cause. Undigested bits of potato were frequently observed in the evacuations. Potatoes when boiled down to a soup were less pernicious, if not altogether harmless. The indigestibility of potatoes, particularly those commonly called *sad* ones *(schliefig)*, has certainly in many instances favoured the outbreak of the cholera. If what is called the ripeness of the potato depends on the perfect conversion of its protéine into starch, we may with justice lay the blame on unripe potatoes: with these, "sad" potatoes stand in the same position. All these circumstances are insufficient in themselves to account for the production of the disease, for which in every case a predisposition appears to have been necessary. Whether this predisposition is attributable to universal terrestrial conditions, as in the disease in potatoes and grapes, is not yet very clear as far as present observations go. What Dr. Schultz says further on the ozone and humidity-contents of the atmosphere has been already noticed.

Health Councillor Dr. Bressler, physician to the ninth medical district, in which one hundred and ninety-five cases occurred, and where Büsching-street, Wine-street, and the front of New King's-gate principally suffered, observes, that the circumstance that many poor were there crowded together does not explain the great number of cases; since in Prenzlau-street and Gollnow-street there are also many overcrowded cottages, and yet the number of cases was here small. The solution of this puzzle is all the more difficult, when it is considered that the houses in Büsching-street, Wine-street, and in front of the new King's-gate, stand much more open than all the rest of the houses in the whole medical district. There can be no question here of confined space and want of fresh air, circumstances which are usually supposed to favour the origination of cholera, and yet there were from four to five times more cases in proportion to the number of inhabitants, than in the narrow Gollnow-street, in which the circulation of air is very much impeded. Dr. Bressler proposes the question, whether under certain circumstances an admission of fresh air may not be prejudicial as actually being the bearer of the miasm? It certainly seems extremely surprising that of the forty streets which compose this medical district, precisely the most openly situate of them were those which were most visited.

In the tenth medical district, in which, in all, there occurred seventy-six cases, the progress of the epidemic being quite undecided, and the cases occurring solitarily, there was, according to Dr. Sieber, no proof of baneful local influences. It was remarked that exactly in the dwellings which were pointed out in the commencement as being unhealthy, there were no cases of cholera at all. Respecting the influence of errors in diet, and cold, Dr. Sieber asserts: I have, as well in this as in previous epidemics, made inquiry in every case to which I have been called, as to whether any errors in diet had preceded the attack; but only very seldom could I find anything of the kind, so seldom, indeed, that I cannot think that diet has any influence whatever. It may, nevertheless, be advisable in future epidemics to make some more general inquiries, because it is precisely a deficiency in food which is likely to keep people in remembrance and in fear of the cholera. Again; colds would seem to furnish a condition peculiarly favourable to the development of cholera. Now, as well as on previous occasions, I have especially noticed that women after certain household operations which are very likely to cause colds, viz., after house-cleanings, and especially after washing-days, have fallen ill in great numbers.

In the first medical district (die Königstadt), where in all there were one hundred and fifty-two cases, several centres of infection having formed themselves, especially in the situation of the Roch-bridge, in the Stadtvoigtei, the King's-wall, and in the house No. 33 in Stralauer-street, and where no decided progress of the epidemic could be traced out. According to the report of Dr. Wolff, the district medical officer, exactly those streets were free from the disease which were the least likely to be so exempt, considering their circumstances, such as Padden-row, little Post-street, little Burg-street, Crown-row, Prob-street, Kalands-court, etc.

Among thirty-eight cases of cholera, in which visits were paid to the dwellings of the patients, nineteen dwelling places were found to be dangerously over-crowded. As exciting causes, Dr. Wolff particularised in eight instances, colds; in four, errors in diet; in three, both colds and errors in diet; in two, mental depression; in eight, neglected diarrhœas; and in one case, infection seemed to be undoubted.

In the Stadtvoigtei prison it never occurred that several persons were attacked in one and the same cell, but all the persons attacked were shut up in different cells along with other prisoners in common, up to the period of their being taken ill.

Health-councillor Dr. Hammer, medical officer of the fifth district, in which there were only twenty-three cases, refuses to recognise local conditions as having any influence in the production of cholera, of which they are so frequently falsely accused; saying that precisely healthily situate and dry places were often very violently visited with cholera. He looks upon debility, errors in diet, colds, and depressing mental affections as predisposing causes. Of his twenty-three cases, it only happened three times that two persons were attacked in one house and one family. One of these (the first in the district) was where a man had taken a sick friend into his house to nurse. This man brought his mother with him from Bernburg to wait upon him, and she also was taken ill. In another instance, two children fell ill, the only assignable cause being that their father had been attending the funeral of a cholera patient.

Towards making out the statistics of the epidemic, it is to be remarked: the total of cases was 1405, and as the population of Berlin is about 446,000, it results that there was only one case of cholera to every 317 inhabitants.

In former epidemics there fell ill of cholera:

| | | |
|---|---|---|
| In 1831 - - - | 1 to 101 | inhabitants. |
| 1832 - - - | „ 382 | „ |
| 1837 - - - | „ 74 | „ |
| 1848 - - - | „ 166 | „ |
| 1849 - - - | „ 75 | „ |
| 1850 - - - | „ 352 | „ |
| 1852 - - - | „ 1804 | „ |

The total of deaths was 940; of recoveries, 465; that is to say, there died 66·9 per cent. of those who were taken ill. This proportion, though the difference is not very great, is more unfavourable than in previous epidemics, with the exception of that of the year 1832, for there died:

| | | | |
|---|---|---|---|
| In the epidemic of 1831 - - | 62·5 | per cent. | of the sick. |
| 1832 - - | 67·2 | „ | „ |
| 1837 - - | 65·7 | „ | „ |
| 1848 - - | 66 2 | „ | „ |
| 1849 - - | 66·2 | „ | „ |
| 1850 - - | 60·0 | „ | „ |
| 1852 - - | 66·8 | „ | „ |

If we compare the proportions of all the epidemics, the fact seems to be more than ever confirmed, that the amount of mortality in cholera, notwithstanding the progress made in medical science, is but little under the control of medicine. The proportion of mortality may in different countries and in different towns appear to differ in some degree, perhaps because the intensity of the disease or the influencing circumstances may differ from one another; but still more probably because the different practitioners who attend or report the cases may not be all alike agreed upon the distinctions to be drawn between cases of true cholera and ordinary cases of "purging and vomiting". It certainly would appear that in Berlin, at any rate, the proportion of deaths in all the epidemics varies very little, and that up to the present time it must be regarded as a law of the disease that the proportion of recoveries to that of deaths is as 1 to 2; and if one were to take the trouble to collect the statistics of all other countries, it is highly probable that the same law would be found to prevail in all alike.

The proportion of deaths to inhabitants in 1853 was 1 to 474.

The cholera deaths in the year 1853 were in the proportion of 1 to 13·23 of the deaths from all causes—the total of these being 12,438.

The deaths from all causes in 1853 give the following per centages: of 12,438 deaths, there were—

| | | |
|---|---|---|
| Still-births - - - - - - | 4·82 | per cent. |
| Debility from birth - - - - - | 3·50 | ,, |
| Gastric and nervous fevers - - - | 4·07 | ,, |
| Inflammatory diseases - - - - | 12·40 | ,, |
| Apoplexy - - - - - - | 9·40 | ,, |
| Spasms - - - - - - | 7·87 | ,, |
| Scarlatina - - - - - - | 1·10 | ,, |
| Dropsy - - - - - - | 4·71 | ,, |
| Consumption - - - - - - | 21·45 | ,, |
| Organic diseases - - - - - | 0·67 | ,, |
| Diarrhœa, purging and vomiting, and dysentery | 4·23 | ,, |
| Cholera - - - - - - | 7·55 | ,, |
| Puerperal fever - - - - - | 0·66 | ,, |
| Cancer - - - - - - | 1·21 | ,, |
| Debility - - - - - - | 4·15 | ,, |
| All other causes - - - - - | 9·21 | ,, |

A very different proportion of deaths was observed in this as in earlier epidemics, between such sick persons as were treated in proper hospitals or sick-houses, and such as were attended in their own dwelling-places.

| | | | |
|---|---|---|---|
| 1. In the cholera hospital of the sanitary commission, after abstraction of such persons as were brought sick of cholera to Berlin from other places, and of such as not having cholera at all were at once dismissed; but, nevertheless, including such as were brought in dead or dying, there were admitted - | 286 | - 180 | deaths. |
| 2. In the small-pox hospital - - | 128 | - 76 | ,, |
| 3. In the hydropathic establishment (Kommandant-street) - - - - | 1 | - 1 | ,, |
| 4. In the Stadtvoigtei lazaretto - - | 1 | - 1 | ,, |
| 5. In the diaconal house of Bethany - | 11 | - 6 | ,, |
| 6. In six different military hospitals - | 21 | - 10 | ,, |
| On the other hand there remained at home | 957 | 666 | |
| | 1405 | 940 | |

Thus there died of those treated in lazarettos, in spite of the difficulties and dangers attending their removal thither, only 61·1 per cent.; while of those who staid at home, 69·5 per cent. died.

The epidemic lasted from the 7th of August to the end of November—nineteen weeks; it took place at the same time of the year, and had about the same duration as in all former cholera epidemics in Berlin. Taking single days we find the following numbers of cases and deaths:

| Days. | August 1853. | | September 1853. | | October 1853. | | November 1853. | |
|---|---|---|---|---|---|---|---|---|
| | Fell ill. | Died. | Fell ill. | Died. | Fell ill. | Died. | Fell ill. | Died. |
| 1 | — | — | 23 | 12 | 26 | 25 | 6 | 5 |
| 2 | — | — | 21 | 14 | 23 | 17 | 4 | 1 |
| 3 | — | — | 17 | 6 | 12 | 14 | 2 | 2 |
| 4 | — | — | 28 | 19 | 12 | 14 | 4 | 3 |
| 5 | — | — | 36 | 15 | 9 | 12 | 2 | 3 |
| 6 | — | — | 18 | 12 | 13 | 4 | 3 | — |
| 7 | 1 | — | 27 | 12 | 6 | 7 | 1 | 1 |
| 8 | — | 1 | 26 | 15 | 16 | 7 | 1 | 3 |
| 9 | — | — | 21 | 11 | 2 | 6 | 1 | — |
| 10 | — | — | 27 | 15 | 9 | 4 | — | 1 |
| 11 | — | — | 34 | 24 | 10 | 7 | — | — |
| 12 | 1 | 1 | 33 | 19 | 14 | 5 | 2 | — |
| 13 | 1 | — | 35 | 19 | 6 | 9 | 2 | — |
| 14 | 1 | 1 | 32 | 24 | 10 | 5 | 1 | — |
| 15 | — | — | 33 | 22 | 10 | 6 | 1 | 2 |
| 16 | 1 | 2 | 41 | 19 | 11 | 5 | 2 | 1 |
| 17 | — | — | 45 | 37 | 12 | 6 | — | — |
| 18 | 2 | — | 37 | 31 | 7 | 8 | — | — |
| 19 | 2 | — | 31 | 27 | 9 | 9 | 1 | — |
| 20 | 5 | 7 | 41 | 29 | 7 | 7 | — | 1 |
| 21 | 1 | 1 | 28 | 27 | 3 | 3 | — | — |
| 22 | 6 | 3 | 28 | 17 | 10 | — | — | — |
| 23 | 3 | 4 | 49 | 27 | 5 | 8 | — | — |
| 24 | 5 | 1 | 56 | 30 | 12 | 3 | 1 | — |
| 25 | 15 | 7 | 35 | 17 | 2 | 7 | 1 | — |
| 26 | 6 | 9 | 39 | 27 | 2 | 1 | — | — |
| 27 | 7 | 3 | 32 | 30 | 1 | 2 | — | 1 |
| 28 | 10 | 4 | 47 | 28 | 4 | 5 | — | — |
| 29 | 13 | 6 | 30 | 27 | 2 | — | — | — |
| 30 | 11 | 10 | 31 | 22 | 3 | 1 | — | 1 |
| 31 | 26 | 11 | — | — | 4 | 3 | — | — |

The epidemic, therefore, increased until the close of the seventh week. From the beginning of the second half of September, it remained at its height for fourteen days, and then steadily declined during ten weeks and a half. Half of the number of cases had occurred by the middle of the seventh week. Half of the number of deaths took place also within the same period, a proof that the epidemic maintained the same malignancy until its termination which it possessed during its increase, a circumstance which the experience of former epidemics in this place had also shown.

Respecting the influence of age and sex, or the number of cases of attack, recovery, and death, the following table gives us some information:

| Ages. | Taken ill. | | Recovered. | | Died. | |
|---|---|---|---|---|---|---|
| | Males. | Females | Males. | Females | Males. | Females |
| Under 3 years | 63 | 57 | 7 | 12 | 56 | 45 |
| 3 to 15 „ | 120 | 127 | 33 | 53 | 87 | 74 |
| 15 30 „ | 137 | 196 | 71 | 90 | 66 | 106 |
| 30 50 „ | 223 | 226 | 72 | 85 | 151 | 141 |
| 50 60 „ | 57 | 77 | 13 | 14 | 44 | 63 |
| Above 60 „ | 50 | 72 | 6 | 9 | 44 | 63 |
| | 650 | 755 | 202 | 263 | 448 | 492 |
| Total...... | 1405 | | 465 | | 940 | |

So that, firstly, among the sick 46·2 per cent. were males, and 53·7 per cent. were females, a proportion which is somewhat in favour of the male sex.

The last census, that of 1852, shows that the civil population of Berlin consisted of 49·7 per cent. males, and 50·2 per cent. females. If we count in the military population, the male population will be found to outnumber the female.

Among the cholera patients there were—

In 1849, 47·5 per cent. males, and 52·4 per cent. females.
„ 1850, 52·0 „ 48·0 „
„ 1852, 55·8 „ 44·2 „
„ 1853, 46·2 „ 53·7 „
The mean proportion of the four epidemics being
48·2 „ 51·7 „

Secondly. The mortality of the male patients was 68·9 per cent.; that of the female patients, 65·1 per cent.

Through this more favourable mortality-proportion on the side of the female patients, the amount of danger to females attacked with cholera in the last epidemic is in a slight degree diminished, so that among the cholera deaths of 1853, 47·6 per cent. were males and 52·3 per cent. females. Compared, however, with the total mortality of the year 1853, this proportion would appear to be highly unfavourable for the female sex, for there died altogether in 1853, 6,510 males and 5,928 females; in other words, 52 per cent. of all who died were males, and 47 per cent. females.

In the epidemic of 1849, the deaths among male cholera patients were 66·5 per cent.; among female cholera patients, 60·0 per cent.

In the epidemic of 1850, the deaths among male cholera patients were 59·8 per cent.; among female cholera patients, 60·2 per cent.

In the epidemic of 1852, the deaths among male cholera patients were 67·4 per cent.; among female cholera patients, 66·0 per cent.

The mortality between patients of both sexes in all these epidemics differs less than in that of 1853; and if we take the year 1849 as giving, on account of the greater extent of the disease, the best measure, the mortality among males appears to be almost equal to that among females, or at any rate only in a very insignificant degree greater.

Thirdly. Among children up to three years of age the proportion of cases and deaths was greater among males than among females, a circumstance which agrees with, and is explained by, the fact that the mortality generally among children is greater with males than with females. At ages above three, cases were more frequent among females, while on the other hand the mortality among males was greater than that among females in the years between three and fifteen and between thirty and fifty.

Fourthly. Of cholera cases, there occurred the following proportions according to the ages of the persons attacked:—

| | | | | | | |
|---|---|---|---|---|---|---|
| Between | 0 | and | 3 | years | 8·5 | per cent. of cases. |
| ,, | 3 | ,, | 15 | ,, | 17·5 | ,, |
| ,, | 15 | ,, | 30 | ,, | 23·7 | ,, |
| ,, | 30 | ,, | 50 | ,, | 31·9 | ,, |
| ,, | 50 | ,, | 60 | ,, | 9·5 | ,, |
| Above | 60 | ,, | .. | ,, | 8·6 | ,, |

These proportions agree pretty nearly with those of 1852, when the per centages at the ages given above were 8, 15, 28·1, 9·4, and 9·4 respectively.

If these proportions be compared with the numbers of the population at these different ages, there being 33·5 per cent. under 16 years of age, 62·4 per cent. between 16 and 60, and only 4·4 per cent. above 60, there would appear to be so unfavourable a proportion for persons above 60 that the danger would actually be double for them.

Fifthly. The mortality among the cholera cases bore the following proportions:—

| | | | |
|---|---|---|---|
| From | 0 to 3 years | 84·1 | per cent. |
| ,, | 3 to 15 ,, | 65·2 | ,, |
| ,, | 15 to 30 ,, | 57·6 | ,, |
| ,, | 30 to 50 ,, | 65·0 | ,, |
| ,, | 50 to 60 ,, | 79·1 | ,, |
| Above | 60 | 87·1 | ,, |

The amount of mortality, as in other epidemics, diminished gradually up to the age of 30, and began to increase again, so that at the greatest age it was greater than in the earliest childhood. We have, therefore, in the case of the aged, to add to the unfavourable proportion of cases this greater proportion of mortality.

The number of cases, as distributed among the different classes of society, is shown in the table appended here.

*Review of Cholera Cases, arranged as they occurred among the different Conditions of Society.*

| Profession. | Families. | Cases. |
|---|---|---|
| Actors (female) | — | 1 |
| Almsfolk: | | |
| Males | — | 1 |
| Females | — | 6 |
| Bakers | 7 | 4 |
| Barbers | 4 | 6 |
| Basketmakers | — | 3 |
| Bookbinders | 1 | 1 |
| Booksellers | — | 3 |
| Braziers | 2 | 1 |
| Brewers | 1 | — |
| Butchers | 5 | 5 |
| Buttonmakers | — | 3 |
| Carpenters | 4 | 7 |
| Carriers | 22 | 19 |
| Chemists | 1 | 1 |
| Chimneysweeps | 2 | — |
| Cigarmakers | 1 | 1 |
| Cloth Shearers | — | 4 |
| Combmakers | 1 | 1 |
| Coopers | — | 2 |
| Copperplate Printers | — | 3 |
| Coppersmiths | 1 | 1 |
| Cotton Printers | 3 | 1 |
| Distillers | — | 1 |
| Domestic Servants: | | |
| Males | 28 | 31 |
| Females | — | 87 |
| Fireworkmakers | 10 | 5 |
| Fishermen | 2 | 2 |
| Furriers | 1 | 2 |
| Gardeners | — | 3 |
| General Traders (*Handelsleute*) | 2 | 22 |
| Gilders | 2 | 2 |
| Gingerbread Bakers | 1 | 1 |
| Glaziers | 3 | — |
| Glovers | 1 | 3 |
| Gunmakers | 1 | — |
| Gymnasiasts | — | 1 |
| Hairdressers | — | 1 |
| Hatmakers | — | 2 |
| Hospitallers: | | |
| Males | — | 5 |
| Females | — | 6 |
| Houseless: | | |
| Males | — | 22 |
| Females | — | 22 |
| Joiners | 18 | 33 |
| Labouring People | 75 | 98 |
| Lacemakers | 1 | — |
| Landed Proprietors | 1 | 4 |
| Letter Founders | — | 2 |
| Locksmiths | 8 | 8 |
| Machinemakers | 7 | 8 |
| Masons | 8 | 11 |

| Profession. | Families. | Cases. |
|---|---|---|
| Medical Practitioners: | | |
| Human | 1 | 3 |
| Veterinary | — | 1 |
| Mechanicians | — | 4 |
| Merchants | 14 | 14 |
| Military | 25 | 17 |
| Millers | 2 | 2 |
| Musical Instru. makers | 1 | 1 |
| Musicians | 3 | — |
| Needlewomen | — | 68 |
| Office Clerks | 19 | 25 |
| Orphan boys | — | 2 |
| Painters | 2 | 9 |
| Paperhangers | 1 | 1 |
| Papermakers | — | 1 |
| Police | 5 | 22 |
| Potters | — | 5 |
| Preachers | — | 3 |
| Printers | 3 | 1 |
| Prisoners (criminal & political): | | |
| Males | — | 9 |
| Females | — | 3 |
| Rentiers | 7 | 11 |
| Saddlers | 3 | 5 |
| Sailors | 30 | 19 |
| Shipwrights | — | 3 |
| Shoemakers | 25 | 46 |
| Sick-nurses (female) | 1 | 1 |
| Slaters or Tilers | 1 | — |
| Smiths | 6 | 6 |
| Soap Boilers | 1 | 1 |
| Stonemasons | 2 | 1 |
| Stone Setters | 1 | 2 |
| Stoolmakers | 12 | 49 |
| Sugar Boilers | 1 | 5 |
| Sword Cutters | — | 5 |
| Tailors | 25 | 46 |
| Tanners | 3 | 6 |
| Teachers | 4 | 5 |
| *Techniker* | — | 1 |
| Tinkers | 3 | 5 |
| Tobacconists | 2 | 4 |
| Turners | 1 | 5 |
| Type Polishers | — | 3 |
| Varnishers | 2 | 2 |
| Victuallers | 1 | 10 |
| Weavers | 1 | 7 |
| Wheelwrights | 1 | 4 |
| Whipmakers | — | 1 |
| Widows | — | 34 |
| Workers in Gold & Silver | 6 | 1 |
| Workhouse People: | | |
| Males | — | 8 |
| Females | — | 21 |
| Writers | 1 | 1 |

This review, as has been observed in earlier epidemics, gives no particular conclusions respecting the predisposition to, or immunity from, cholera, of this or that condition of life. The surprisingly small number of cases among military persons was as gratifying in this as in earlier epidemics. Police-officers also, notwithstanding the more arduous nature of their service, were almost equally favoured. In both these classes something must be attributed to the great care taken of them by their superior officers, and the immediate medical attendance available for them. Medical practitioners and attendants on the sick were quite untouched by the cholera mortality of the present epidemic.

The local spread of this epidemic scarcely differed from that of earlier ones. In this year also it was near the older parts of the town—the Friedrich-Wilhelmstadt—that the disease was most severe; in return for which the Friedrichstadt, especially that portion of it situated westward of Leipsic-street, suffered but little. A distinctly-marked progress of the disease through the town, made itself manifest no further than that the first cases showed themselves in the seventh and eighth medical districts on the right bank of the Spree. When the disease had after fourteen days become more rife, it was spread pretty equally all over the town, the westernmost parts, however, remaining almost exempt. The cessation of the epidemic, which took place gradually in November, was tolerably uniform in all parts of the town.

In proportion to the number of inhabitants (in the different streets) the number of cases were remarkable, especially Fish-street, old James-street, Charles-street, King's-wall, Albert-street, Shoemaker-street, Breslau-street, and Wall-street.

The numbers of the sick in the Milk-market, in Alexander-street, and Undertree-street, appeared extraordinary in themselves, but they are not so when compared with the number of inhabitants, because in them are found the stadtvoigtei prison, the work-house, and the charity hospital. As far as regards the above-named streets, which are distinguished for the greatest mortality, it is to be observed that they also suffered most in former epidemics, and were such as either, like Fish-street, King's-wall, old James-street, Breslau-street, and Wall-street, combine, besides their neighbourhood to the water, narrow dwellings in high houses, bad courtyards, a very numerous and poor population, and a great variety of trades, frequently of a not very cleanly kind; or, like Charles-street, Shipbuilder's-pier, Albert-street, and Shoemaker-street, in the newly-built Friedrich-Wilhelmstadt in the neighbourhood of the Spree or of the Panke, lie on a soil which has recently been formed out of a marshy, boggy piece of land.

With respect to cases of sickness and death occurring in different houses, it is not without interest to remark that:—

1. In houses, which in former epidemics had been entirely free from cholera, 581 persons were attacked, of whom 374 died.

2. In houses in which in previous epidemics cholera cases had occurred, 741 were attacked, of whom 510 died.

3. Besides these, partly houseless, partly in boats, partly in barracks, 83 persons fell ill, and 56 of them died.

It appears, therefore, that taking single houses into consideration, 43·9 per cent. of the cases occurred in houses previously exempt, and 56·1 per cent. in houses which had been visited by the disease in previous epidemics. But the mortality in the former class of cases amounted to only 64·3 per cent. of them, while in those previously visited it amounted to 68·8 per cent.

The houses in which the greatest number of cases occurred (five and upwards), were the following:—

| | Cases. | | Deaths. |
|---|---|---|---|
| The workhouse in Alexander-street - - | 31 | ,, | 17 |
| The Charité Hospital in Undertree-street - - | 25 | ,, | 10 |
| The Kottwitz poorhouse in Alexander-street - | 19 | ,, | 13 |
| The new Hospital in Wall-street - - - | 12 | ,, | 11 |
| The Stadtvoigtei in the Milk-market - - | 12 | ,, | 7 |
| The House No. 17 in Charles-street - - | 11 | ,, | 10 |
| No. 14, Wine-street - - - - | 9 | ,, | 5 |
| ,, 38, Fish-street - - - - - | 7 | ,, | 5 |
| ,, 30, Charles-street - - - - - | 7 | ,, | 7 |
| ,, 5, Milk-market - - - - - | 7 | ,, | 3 |
| ,, 18, Albert-street - - - - - | 6 | ,, | 4 |
| ,, 92, Garden-street - - - - - | 6 | ,, | 5 |
| ,, 80, Albert-street - - - - - | 6 | ,, | 4 |
| ,, 41, Kranston-street - - - - - | 6 | ,, | 4 |
| ,, 16, Old Leipzig-street - - - - | 6 | ,, | 2 |
| ,, 15, Bush-street - - - - - | 5 | ,, | 5 |
| ,, 22, New Green-street - - - - | 5 | ,, | 2 |
| ,, 23, Neuköll on the Water - - - | 5 | ,, | 3 |
| ,, 7, Winemaster-street - - - - | 5 | ,, | 3 |
| ,, 19, Shoemaker-street - - - - | 5 | ,, | 1 |
| ,, 102, Farmer-street - - - - - | 5 | ,, | 0 |

In the symptoms of the disease, and in the *post mortem* appearances presented by those who died of it, the epidemic of 1853 shewed no difference from what had been observed in previous epidemics. And, as it would seem, but little improvement has been made in the treatment. We will presently lay before our readers the observations made in the state hospital on the effects of the different plans of treatment which were tried there.

The sanitary police regulations were in this epidemic carried out in the same way as in previous ones. The sanitary commission, which was established in accordance with the regulations of the 8th August, 1835, as soon as the outbreak of cholera in Copenhagen, Dantzic, and Stettin was made known, turned its attention to the re-establishment of cholera hospitals. In order to provide the town with them in all directions, three hospitals were established in different districts. In the new hospital on the Weisenbrücke, in which there was already a medical establishment, of which the necessity was constantly felt, cholera wards were opened. The Charity-Direction opened a second institution, in accordance with an understanding come to with the commune, in the small-pox hospital belonging to the Charity, and it was agreed that it should be under the direction and superintendence of the physician of the Charity-Direction. The third institution was established near the Hallisch Thor, in an old barrack which was readily given up for the purpose by the military authorities, but was not opened, because in the course of the epidemic there never arose any necessity for it.

Under the jurisdiction of the sanitary commission, therefore, there was only the *Heilanstalt* in the new hospital, and here, in the beginning, a separate side-building was used exclusively for the cholera patients. When at a later period this place no longer supplied sufficient space, and was also unsuitable for the cold season of the year, it became necessary to make use of a portion of the main building. This portion was made perfectly separate from the rest of the building occupied by ordinary patients, so that there was no communication whatever between them, as had been the case in former epidemics. The anxiety felt by the trustees of the new hospital lest the general patients should be in any danger from the neighbourhood of the cholera patients, was fortunately not justified.

In addition to the establishment of these hospitals, the university commission so far took care of the medical treatment of the poor cholera patients who remained in their own houses, that authority was given to all the resident medical men to furnish, at the expense of the commune, suitable medicines for all poor cholera patients, in order that no delay in the treatment of a case should arise through the difficulty of procuring the official physician when wanted. Through the limited spread of the disease, this arrangement proved to be quite sufficient, so that there was no necessity for the appointment of professional night-guards, or of an increase in the number of the official physicians, which last measure would be highly expedient in the event of a greater spread of the epidemic; for the successful treatment of cholera especially requires that the patient should be frequently visited, for which in a great epidemic the present number of parish doctors would be quite insufficient.

Next after these measures which were required for the treatment of the sick, it became the duty of the sanitary commission to take every possible care with a view of preventing the spread of the disease. Remembering that the course of former epidemics proved the miasmatic nature of the disease just as much as the development of contagion under certain circumstances did not admit of dispute, the sanitary commission promoted, as far as they could, disinfection, so that 227 disinfections were effected in 151 dwelling-houses by the disinfectors of the sanitary commission, while in the remaining cases they were partly carried out by the parties concerned, and partly expressly refuted by them. The commission likewise ordered the immediate removal of corpses out of small dwellings into dead-houses which were provided for the purpose, prohibited the assembling of mourners in the dead-houses, watched over the traffic in the markets, so as to put a check on the sale of unripe fruits and other articles of diet likely to injure the health, attended to the cleansing of the streets, and to the removal of all nuisances from public institutions, likely to predispose to the disease (in the workhouse, and the Stadtvoigtei prison, for example), prohibited the annual mud-clearing from the branches of the Spree and from the canals, and took care that, by a careful supervision of the sluices, the flow of water should be properly regulated.

The numerous complaints made of the condition of the river Panke, although before the epidemic public attention had already been directed to it, led to frequent thorough local inquiries. But it was precisely this year that the condition of the river was at least such as to cause it to be considered dangerous to health, or the reasonable cause of the frequent cholera cases occurring in its neighbourhood. Much more was there reason to fear for the effects of a pond near the Panke, situated in the garden of the Veterinary School, a piece of almost stagnant water in the midst of a built-up portion of the town, and for the future drying-up of which orders were given. Besides this condition of the Panke, the wells in the house, No. 30, in Charles-street, in Friedrick-Wilhelm stadt, near the Panke, attracted the public attention, and were especially accused of having contributed to the spread of the disease, because in that house the whole of a family belonging to the upper classes, and consisting of six persons, died of cholera. An inflammable gas escaped from the surface of the water of one of these wells. A commission, consisting of Dr. Magnus, Professors Ehrenberg and Rammelsberg, and Branddirector Scabell, appointed to investigate this phenomenon, found the source of this exhalation, which consisted of marsh-gas (light carburetted hydrogen) in a ditch, which was connected with the above-mentioned neighbouring pond of the Veterinary School, passed under the house, No. 30, in Charles-street, and communicated with the shaft of the well through a defective part of its wall. Since the reparation of this defective portion of wall the phenomenon has ceased. The water of this well, which, chiefly by the smell, but not by the taste, showed traces of marsh-gas, had been used without any disadvantage by the inhabitants of the house; only the above-named family had constantly had their drinking-water fetched from another well. But this house was not at all especially visited; in a house lying obliquely over against it, No. 17, Charles-street, on the other side of the Panke, there died as many as ten persons of cholera.

*The cholera-wards in the New Hospital.*—These wards were opened on the 24th of August, and closed on the 30th of November. During this period there were brought in 290 persons; after deducting from these 2 who were sent away as not being ill with cholera, 2 who were brought in dead, and 11 who were moribund when brought in, there remain 275. Of this number were cured 107, or 38·50 per cent. and there died 188, or 61·50 per cent. Including the dead and dying when brought in, there were 141 persons of the male sex and 149 of the female. Of the former were cured 52, and died 89; of the latter were cured 57, and died 92. The ages of the patients admitted, excluding the two which were not ill of cholera at all, were as follows :—

| | | | | | | |
|---|---|---|---|---|---|---|
| Between 1 and 10 years | 28. | Of these died | 13 | 46 | per cent. |
| ,, 10 ,, 20 ,, | 25. | ,, | 8 | 32 | ,, |
| ,, 20 ,, 30 ,, | 88. | ,, | 42 | 47 | ,, |
| ,, 30 ,, 40 ,, | 53. | ,, | 36 | 69 | ,, |
| ,, 40 ,, 50 ,, | 38. | ,, | 28 | 73 | ,, |
| ,, 50 ,, 60 ,, | 37. | ,, | 35 | 94 | ,, |
| ,, 60 ,, 70 ,, | 9. | ,, | 9 | 100 | ,, |
| Above 70 ,, | 10. | ,, | 10 | 100 | ,, |

The length of time they were in the hospital was

| | | | | | | |
|---|---|---|---|---|---|---|
| Less than 1 hour with | 10 persons, | of these | 0 | recovered, | 10 | died. |
| From 1 to 4 hours with | 11 | ,, | 0 | ,, | 11 | ,, |
| From 4 to 8 hours with | 17 | ,, | 0 | ,, | 17 | ,, |
| From 8 to 12 hours with | 14 | ,, | 0 | ,, | 14 | ,, |
| From 12 hours to 3 days with | 74 | ,, | 1 | ,, | 73 | ,, |
| From 3 to 6 days with | 47 | ,, | 10 | ,, | 37 | ,, |
| From 6 to 8 days with | 18 | ,, | 11 | ,, | 7 | ,, |
| From 1 to 2 weeks with | 49 | ,, | 41 | ,, | 8 | ,, |
| From 2 to 4 weeks with | 39 | ,, | 38 | ,, | 1 | ,, |
| Above 4 weeks with | 9 | ,, | 9 | ,, | 0 | ,, |

The patients with very few exceptions belonged to the so-called working-classes, and came partly out of private houses, partly out of public institutions, viz., from—

| | |
|---|---|
| The Workhouse . . . | 30 |
| Friedrich's Orphan House . . | 1 |
| The New Hospital . . . | 12 |
| The Stadtvoigtei Prison . . | 12 |

With regard to the patients from the New Hospital, we have to remark that the cholera wards were in that hospital. In the first days after the opening of these wards they were contained in a neighbouring building, entirely separated from the hospital proper. During this period eleven hospital patients were attacked with cholera. The increase in the number of patients in the wards, and the unsuitableness of the locality, for the cold season of the year rendered it necessary to effect a removal on the 7th of September, and six wards of the hospital-proper were given up to cholera patients, in such a way, however, that the cholera part had its own separate entrance, and was separated from the portions left for the other patients by boarded partitions covered with reeds and lime-wash. The courtyard of both institutions remained common. After this removal only one other hospital patient was attacked with cholera. For the rest the whole number of the hospital patients amounted in the beginning to one hundred and ninety-eight; afterwards, when from the overflowing of the cholera institution, the removal of some of the hospital patients to the workhouse became necessary, one hundred and thirty-nine. Not one of the patients removed to the workhouse was there taken ill of cholera.

When we reflect that the hospital patients are the sick and infirm of the lowest class, persons who, from their constitution of body, would seem to be especially liable to be attacked with cholera; that, as a further predisposing element, the situation of the New Hospital is in Wall-street, a portion of the town which was much visited in former epidemics; and that in earlier epidemics, as well the inhabitants of the workhouse generally as also in an especial degree the hospital patients, were attacked in proportionately greater numbers, the fact so important in the question of the contagious nature of the disease, which was already ascertained from previous epidemics in Berlin, that the immediate neighbourhood of a cholera hospital is not dangerous to the neighbouring population, will appear to be abundantly confirmed.

Even among the persons who were in the most immediate and lasting intercourse with the patients—the physicians and nurses, there occurred no cases of cholera which could be traced to infection. Solitary cases of slight diarrhœa occurred among the attendants, which showed no characteristic marks of cholera, and very quickly got well again. Two assistant physicians were certainly taken ill on one and the same day with a tolerably violent purging and vomiting, but there was every reason to attribute this circumstance to some other cause than infection. Only one individual among the servants of the establishment fell ill and died of cholera, and this was the porter, who lived in a little house to himself, and was never in any kind of contact with the patients.

The cases were, as is always the case in cholera hospitals, almost entirely of the most intense kind; because in slighter cases and in the commencement of the attack, the resolution to leave their dwelling is seldom conceived and carried out. With patients comming out of prisons and hospitals this rule does not hold; but then the subjects are here so weakly that they can scarcely oppose any resistance to the attack, which with them usually runs through its course with extreme rapidity. One hundred and thirty-five persons were already pulseless when admitted; of these only twenty-three recovered; the remainder died. In a very large proportion, the pulse was sinking when they were brought in.

The symptoms of the disease were, in proportion to the severity of the cases, the same as in previous epidemics, and as they had been especially described in the published notices of Leubuscher,

Reinhard, Guterbock, &c. As the most characteristic symptom of general cholera, the suppression of the secretion of urine, was observed; while in cases of dysentery with vomitings, during the intestinal evacuations, this secretion ceases, but then again returns. In genuine cholera cases it first appeared several days later, if death did not earlier put an end to the matter. The urine was then commonly albuminous; but a decisive prognostic sign of a consecutive stage of typhus could not satisfactorily or with certainty be deduced from the amount of albuminuria. With regard to intestinal evacuations and cramps, there was nothing new to be learned. The stage of reaction offered this peculiarity, that in a comparatively small number was it accompanied by the well known symptoms of typhus fever: altogether, this occurred in only thirty-two cases, of which twenty were fatal, and twelve recovered. Pneumonic symptoms, frequent though they were in former epidemics, were this time extremely rare, and, when they did show themselves, admitted of a more favourable prognosis than before. As favourable signs in the reactive stage, the exanthems were remarkable: these were observed in seven patients, and indeed without any exception in the form of urticaria. As a rule, this urticaria first appeared when the patient was already recovering. In some cases, the symptoms of a light gastric fever immediately preceded the outbreak of the eruption. These patients all recovered, with the single exception of one who had parotitis at the same time. Altogether, this last affection appeared in three patients during the reactive stage, ended in suppuration, and the prognosis was unfavourable: only one recovered; the others died.

*Post mortem* examinations of those who died of cholera offered nothing unusual.

In the medical treatment of the cholera, a search after specific remedies is frequently rejected as irrational. Nevertheless, no physician can be said to be wholly free from a specific treatment of the disease. He who considers the indication to be to rouse the sinking powers by means of stimulants, and who uses with that view ammoniacal medicines without camphor, &c., or camphor without ether, &c., or ether, or *carbo trichloratus* (chloroform?), &c., is exactly seeking for a specific remedy against the disease. Is it then really so objectionable to strive after a specific mode of treatment in cholera?—a disease which carrries along with it so very specific a character, which cannot be regarded as the necessary consequence of a pathological process gradually developing itself in the individual organism, and peculiar to it, but rather by virtue of an external foreign potency—be it contagion or be it miasm—attacks the human subject without the slightest perceptible predisposition on its part, almost suddenly, and with a sameness of symptoms such as few diseases possess, so that it most resembles a poisoning,—such a disease may surely be met by a decided, *specific* method of treatment. It does not hence follow that the physician should blindly experimentalise, or should try perhaps all the articles in the Materia Medica, from A to Z, until he hit upon the right one; but it does follow that the physician, knowing that as yet no specific has been found in the remedies hitherto used, should not rest satisfied with the method used up to the present time, but should attempt a rational plan, that is to say, by attending to the indication which a general knowledge of medicine and his own experience will suggest. On these principles the proceedings in the cholera hospital were conducted: not only were many different remedies tried, but several were used, one after another, on one and the same patient. It was especially thought necessary, when once convinced of the inutility of the medicine in the individual, to avoid continuing the use of it till the patient died, merely because it had been ordered, and because no better remedy was known. These are things which theoretically every physician will allow, but which in practice are very often wanting; and especially in private practice, where the physician, as a rule, cannot see his cholera patient either continuously or often enough, there must be a deficiency, without any fault on his part. But hospital practice affords better opportunities, with the constant supervision of the sick; and hence perhaps it may have arisen that, in spite of the unfavourable effects likely to result from the transport and removal of the patients, the mortality is smaller in hospitals than in the town generally. In a hospital, it is practicable to decide on the exact moment when the inutility of a remedy becomes manifest, and it becomes expedient to look out for another. In other respects, the results of the treatment in the cholera institution were not more favourable than they were in former epidemics in hospitals in this place. A satisfactory procedure has not been found out. Although we may not be able to lay down a method by which every cholera patient may be cured, we ought nevertheless to be able to save all those cases which may not yet have arrived at the stage of asphyxia. But this we have not succeeded in doing. What was the effect of different plans of treatment, with a view to a cure, and also with reference to the cases passing into a typhoid stage, is shown in the following statistical report, in which, as far as possible in such a summary, notice is taken of the stage of the disease.

With reference to the medicinal means employed we have the following proportions:

1. With cold wet envelopings of the body, renewed every half-hour, 34 patients were treated; 8 recovered, and 26 died; or 76·4 per cent. The eight cases saved were asphyxiated. In one of them, acetate of copper was also used; in another, ammonia; and in a third, camphor.

2. With camphor, 28 were treated; of these, 9 recovered, and 19 died; or 67·8 per cent. Among the 9 cases which recovered, 3 were pulseless; 3 with sinking pulse; and 3 of a milder kind. In addition to the camphor, 3 patients took acetate of potash; 3, Dover's powder; and 1 ammonia.

3. With nitrate of silver, 21 were treated; of these, 10 recovered, and 11 died; or 52·3 per cent. Among these were 16 children and 5 adults; all the last died. Among the cases saved, 3 were already pulseless; 2 with sinking pulse; and 5 of a slighter kind.

4. With extract of nux vomica, three grains in six ounces of water, one tablespoonful every quarter, every half, or every hour, 52 were treated; of these, 22 recovered, and 30 died; or 57·6 per cent. Of the 22 saved, 8 were pulseless; 3 with sinking pulse; and 11 were slighter cases. Ten patients used also tincture of acetate of iron, and one had acetate of zinc.

5. With tincture of the acetate of iron by itself, 10 were treated; 6 died, and 4 recovered; the mortality, therefore, being 62·5 per cent. Among the saved, 1 was pulseless.

6. With tincture of acetate of iron and tincture of opium, 8 were treated; of whom, 3 recovered, and 5 died; or 62·5 per cent. Those saved were all slight cases.

7. With tincture of acetate of iron, alternately with tincture of stramonium and acetate of soda, 48 were treated; of whom, 13 recovered, and 35 died; or 72·9 per cent. Of those saved, 5 were pulseless, and 5 with sinking pulse.

8. With phosphorus, 5; 1 of whom recovered and 4 died; 80 per cent. The one who recovered had a sinking pulse, and had camphor as well as phosphorus.

9. With acetate of copper, 7; 1 recovered and 6 died; or 85·7 per cent. The first case was also treated with cold envelopings.

10. With carbonate of ammonia, 10; of whom, 3 recovered and 7 died; or 70 per cent. Among those who recovered, 2 were severe cases.

11. With acetate of zinc, 4; of these, 1 recovered, and 3 died; 75 per cent. The first had besides, extract of nux vomica.

12. With tincture of guaiacum, 7; all of whom died.

13. With partial cold envelopings, 5; who all likewise died.

14. With cyanate of zinc and iron 2, who both died.

15. With nitrate of strychnia 1, who died.

16. With tincture of iodine 5 cases, of which 2 recovered and 3 died, or 60 per cent. The two saved were pulseless.

17. With strong liquid ammonia (a few drops on ice every quarter of an hour) 6, of whom 1 recovered and 5 died, or 83·3 per cent. In the first case cold envelopings were also used.

18. With liquid hydrosulphate of ammonia, 5; of whom 1 recovered and 4 died, 80 per cent. The first case was also treated with cold wet cloths applied to the body.

19. With acetate of soda alone 26 cases treated recovered, 25 of which were light ones, and only 1 (in a female aged 54) was asphyxiated.

20. With acetate of lead and opium with camphor 1 asphyxiated case was treated and recovered.

With all the above-named means, with the exception of the cold envelopings, warm baths and continual frictions and sinapisms were also used. In almost all the cases, on account of the oppression of the chest which was present, blood was abstracted by means of cupping glasses, and very frequently, to the great relief of the patients, friction with veratrine ointment on the pit of the stomach and over the sacrum.

Among all the cases treated 32 passed into the typhoid stage, of whom 12 recovered and 20 died, or 62·5 per cent. With regard to the treatment employed before the appearance of typhoid symptoms, we have the following account:—

After the use of camphor, typhoid fever occurred 3 times. Of these 2 cases recovered, and 1 died—33 per cent.

After the use of carbonate of ammonia, typhoid fever occurred once, and the patient recovered.

After the use of tincture of iodine, typhoid fever occurred in 1 instance, and terminated fatally. The same circumstance occurred after the use of tincture of acetate of copper.

After the use of nitrate of silver, 2 cases of typhoid fever occurred, 1 of which had a fatal termination, the other recovered.

After the use of cold wet cloths, there occurred 4 cases of typhoid fever, 3 of which recovered, while the other ended fatally.

After the use of acetate of potash, there were 7 typhoid cases, 6 of which had a fatal termination—85 per cent.

After the use of tincture of acetate of iron and tincture of stramonium, there were 5 cases of typhoid fever, 4 terminating fatally—80 per cent.

After the use of extract of nux vomica, 8 typhoid cases occurred, of which 5 ended fatally—62 per cent.

The typhoid fever was treated with cold spongings and local bloodlettings, and

| | | | | | | |
|---|---|---|---|---|---|---|
| In 12 | cases with | nitrate of potash; | of these | 4 | died—33 | per cent. |
| 5 | ,, | acetate of potash | ,, | 4 | 80 | ,, |
| 5 | ,, | chlorinated water | ,, | 4 | 80 | ,, |
| 2 | ,, | calomel | ,, | 1 | 50 | ,, |
| 3 | ,, | tincture of acetate of iron | | 2 | 66 | ,, |
| 3 | ,, | mineral acids | ,, | 3 | 100 | ,, |
| 1 | ,, | camphor | ,, | 1 | 100 | ,, |
| 1 | ,, | acetate of zinc | ,, | 1 | 100 | ,, |

After the experiments conducted in the Institution, of all the medicines tried the spirituous extract of nux vomica deserves the greatest attention. It constituted indeed a means, the effect of which in restoring a sinking pulse was quite unmistakeable. The pulse would give way whenever the medicine was stopped, and came again as soon as it was renewed. Nevertheless, it was far from establishing a cure in all cases. In very numerous instances, the pulse gave way in spite of the continued use of the medicine, and the patient died without having passed into the stage of reaction. In the cases in which reaction came on, this reaction was seldom connected with typhous symptoms; for although twentytwo patients recovered who took the extract, typhoid fever occurred in only eight cases in which this medicine had been given.

*Au reste*, it was necessary to go to work very cautiously in the administration of extract of nux vomica, as otherwise symptoms of strychnism were apt to develope themselves. Commonly, three grains in six ounces of water ("when taken to be well shaken") were ordered; and of this mixture, according to the urgency of the symptoms, a tablespoonful was given every quarter of an hour, every half hour, every hour, or less frequently. Frequently nine grains and more were necessary to establish such an amount of reaction that the medicine could be altogether set aside. In some solitary cases, however, traces of strychnism would show themselves before this quantity was taken.

The efficacy of the latterly much belauded acetate of soda was confined to the slighter cases, and in them was indeed surprising. But in such cases, one can never be sure whether they by other means, or even without medical treatment at all, had become asphyxiated. In cases where acetate of soda was useful, there arose a strong perspiration; in all severe and truly asphyxiated cases of cholera a crisis never takes place through a perspiration. With a very favourable result in one case of asphyxiated cholera acetate of lead with opium and camphor was employed. This good result, although a slight evidence of the specific effects of lead was induced, would have led to the trial of this medicine in other similar cases, if this one had not happened to be the last severe case brought into the hospital.

In the treatment of the consecutive typhoid fever it appeared that the mineral acids had not a favourable effect; at any rate they afforded no results in the first cases that occurred. Other remedies were then had recourse to, of which the nitrate of soda gave the most favourable results, even in the most severe cases.

Lastly, the use of nitrate of silver in the asphyxiated stage must be mentioned; with children it acts as a true specific; with adults, according to the experience of the present year, it was completely useless in the hospital.

[Our excellent German neighbours are certainly very bold practitioners, and do not lose their character of being "speculative", in the history of the treatment of cholera pursued in Berlin. To our minds, the various plans of treatment carried out prove nothing but the negative facts that they were all useless, and need not be tried again. In some cases, such information is valuable, especially when derived from experiments based on rational principles, and having some specific end in view. The experiments above related are, however, too purely empirical and venturesome to be favourably considered, even as affording negative evidence.]

